# Septic Arthritis of the Pediatric Wrist: A Case Report and Review of the Literature

**DOI:** 10.7759/cureus.7444

**Published:** 2020-03-28

**Authors:** Jeremy M Silver, William Hennrikus

**Affiliations:** 1 Orthopaedic Surgery, Penn State Milton S. Hershey Medical Center, Hershey, USA

**Keywords:** septic arthritis, wrist, pediatric orthopaedics

## Abstract

Septic arthritis of the wrist in pediatric patients is a rare diagnosis and is not well described in the literature. We present a case of a 4-month old patient with monoarticular septic arthritis of the wrist treated with surgical drainage and antibiotics. Although a rare diagnosis, septic arthritis of the wrist should be considered in patients with pseudoparalysis of the upper extremity and systemic signs of inflammation. Prompt diagnosis and treatment is critical to prevent permanent damage to the joint. Further data is needed to describe the epidemiology, microbiology, diagnostic findings and treatment of septic arthritis of the pediatric wrist.

## Introduction

Septic arthritis of the wrist in the pediatric population is a rare diagnosis. To our knowledge, there are only three case reports of septic arthritis of the pediatric wrist [1-3]. Acute septic arthritis in a pediatric patient can present in a variety of ways, which can make diagnosis difficult. While there is data regarding the more common pediatric septic arthritis of larger joints, the epidemiology, microbiology, diagnostic criteria and outcomes of septic arthritis of the wrist are not well described. We present a case of a 4-month old with monoarticular septic arthritis of the wrist treated with open surgical drainage and antibiotics.

## Case presentation

A previously healthy 4-month old male was transferred from an outside hospital for a two-day history of fever and difficulty moving his right arm. There was no history of trauma, insect bites or recent travel. Upon examination, there was mild tenderness and swelling of the right wrist and hand, most notably on the dorsal aspect. The right elbow and shoulder were non-tender with full range of motion. Initial labs showed an erythrocyte sedimentation rate (ESR) of 31 mm/hr, C-reactive protein (CRP) of 2.45 mg/dL, and white blood cell (WBC) count of 11 x 103/mcL. The patient was afebrile on admission, although a review of outside records showed one temperature of 101.4°F before transfer. X-rays of the right upper extremity showed no evidence of fracture and were not concerning for osteomyelitis.

**Figure 1:**
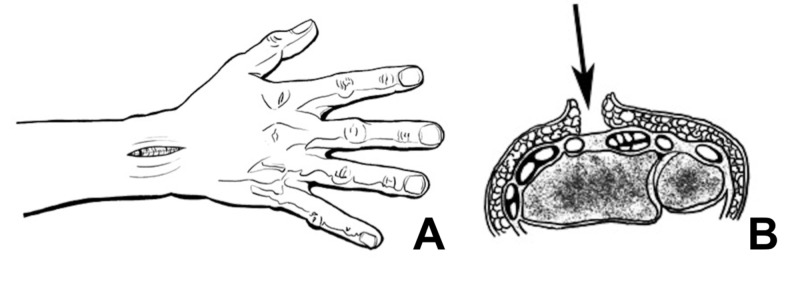
Anteroposterior (A) and axial (B) artist renditions depicting the dorsal approach of the wrist between the 3rd and 4th extensor compartments Illustrations provided with permission by Julia Hennrikus.

After initial examination, the case was discussed with the patient’s parents and consent was obtained for aspiration of the right wrist with potential irrigation and debridement (I&D). He was taken to the operating room and placed under general anesthesia. Aspiration of the dorsal aspect of the right wrist joint yielded 1 mL of pus fluid that was sent to the lab for Gram stain, anaerobic and aerobic cultures. Next, a 2-cm incision was made over the dorsal wrist between the third and fourth extensor compartments (Figure 1). The extensor retinaculum was incised and the extensor tendons were mobilized. Dissection was continued down to the joint capsule and open I&D of the joint was performed with approximately 200 mL of sterile saline. The capsule was left open but the skin was closed, and the patient was placed in a soft splint. The patient tolerated the procedure without issue.

**Figure 2:**
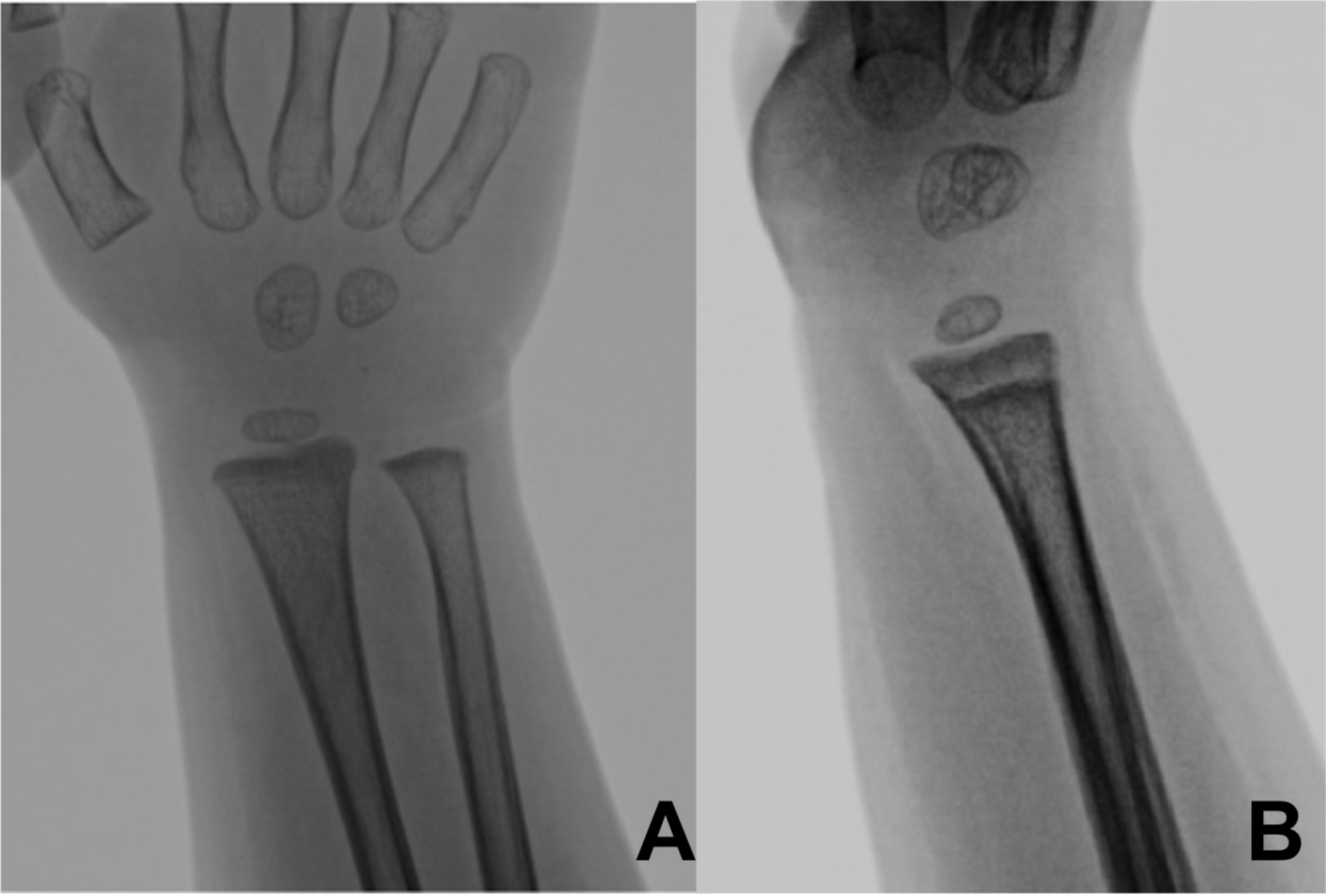
Anteroposterior (A) and lateral (B) radiographs of the wrist two years postoperatively with no evidence of bony abnormalities or growth arrest

Empiric coverage with IV vancomycin and cefazolin was started. On post-op day two, lab work showed an ESR of 29 mm/hr, CRP of 1.73 mg/dL and intra-operative cultures grew methicillin-sensitive Staphylococcus aureus (MSSA) with abundant white cells. Antibiotic treatment was changed to IV nafcillin. On post-op day five, the ESR was 16 mm/hr, CRP 0.56 mg/dL, and the patient was transitioned to oral cephalexin and rifampin. On post-op day seven, the patient was discharged home on oral antibiotics for a total of four weeks. The patient remained afebrile during the course of the hospital stay and was kept in a soft splint for a total of three weeks. Follow-up appointments at two weeks and three months after discharge showed a well-healing scar and a normal CRP. Follow-up at one and two years after discharge confirmed no limitations of the right upper extremity with X-ray confirmation of a well-healed wrist joint with open growth plates and no bony abnormalities (Figure 2).

## Discussion

Septic arthritis of the wrist in the pediatric population is a rare diagnosis. To our knowledge, there have only been three reported cases of pediatric septic arthritis of the wrist in the literature. Wegner et al. in 2017 reported a case of polyarticular arthritis of the left wrist and right knee due to Streptobacillus moniliformis from a rat bite [1]. A retrospective chart review of 40 pediatric patients with septic arthritis by Caksen et al. in 2000 demonstrated only one case of septic arthritis of the wrist [2]. Rahman et al. in 2016 reported a case of septic arthritis of the hand and wrist in an 8-year-old girl due to group A Streptococcus pyogenes treated with surgical drainage [3]. We have presented a case of monoarticular septic arthritis of the wrist in a 4-month old patient successfully treated with open surgical drainage and antibiotics.

The differential diagnosis of pediatric patients presenting with pseudoparalysis of the upper extremity is broad. While other infectious etiologies like Lyme disease may be more common, particularly in endemic areas, septic arthritis should be considered as it can result in permanent joint damage with a delay in treatment. Septic arthritis can occur in any joint but more commonly involves the joints of the lower extremity in the pediatric population. Previous studies have shown an overall incidence of acute septic arthritis in the pediatric population between 2 and 13 out of 100,000 in developed countries [4,5]. Of these cases, roughly 80% involve the knee, hip and ankle with Staphylococcus aureus being the most common organism [4-6]. Most cases are caused by hematogenous spread of bacterial pathogens while others can be caused from direct spread via trauma or infection from other neighboring tissues [7]. Prompt diagnosis and treatment is necessary to prevent permanent joint damage and growth plate involvement. Damage is mediated by both host immune response and bacterial toxins in which high cytokine concentrations result in the release of metalloproteinases and other collagen-degrading enzymes [8].

The initial work-up of suspected septic arthritis should include a thorough history, physical exam and labwork, including a complete blood count with differential, ESR, CRP, blood culture, and synovial fluid analysis [5]. Particular emphasis should be placed on obtaining blood cultures as the results will help guide antibiotic treatment. While both ESR and CRP have been shown to be sensitive markers of childhood bone and joint infections, CRP normalizes faster and should be used to monitor recovery [7,9].

Treatment of septic arthritis in pediatric patients begins with prompt diagnosis, surgical drainage, and initiation of antibiotics to prevent permanent damage to the joint [4-7,10]. Empiric antibiotic coverage should be initiated as soon as blood cultures and synovial fluid samples are collected and should include MRSA coverage in endemic areas [4]. Recent studies have shown that short courses of parenteral antibiotics (2-4 days) followed by transition to oral therapy are sufficient to treat uncomplicated cases of septic arthritis [4-6,10]. Specifically, the combination of clinical improvement and a significant reduction in CRP can be used to guide the transition from IV to oral antibiotics [11]. In this case, the patient was treated with five days of IV antibiotics and was transitioned to oral after clinical improvement and a reduction in CRP from 2.45 mg/dL on admission to 0.56 mg/dL on post-op day 5.

The most appropriate surgical treatment of septic arthritis remains open arthrotomy [4,6]. Although arthroscopic I&D has been shown to have success in some cases, the indications for open vs. closed surgical management are not clearly defined in the literature [4,5]. Furthermore, the small joint space in pediatric patients limits the feasibility of arthroscopy.

Septic arthritis of the wrist in the adult population is better described in the literature but there are no consensus diagnostic criteria and the true incidence remains unknown [12,13]. One recent retrospective chart review by Jennings et al. in 2017 demonstrated a 1.5% incidence of septic arthritis of the wrist in patients presenting with a swollen, painful wrist without trauma [13]. The most common organism of septic wrist in the adult population is also Staphylococcus aureus [12].

## Conclusions

Septic arthritis of the wrist in pediatric patients is a rare diagnosis but should be considered in any patient presenting with swelling, pain or difficulty moving the wrist and systemic signs of inflammation. We present a case of monoarticular arthritis of the wrist in a pediatric patient successfully treated with appropriate antibiotic use and surgical drainage.
